# Comparability of Red/Near-Infrared Reflectance and NDVI Based on the Spectral Response Function between MODIS and 30 Other Satellite Sensors Using Rice Canopy Spectra

**DOI:** 10.3390/s131216023

**Published:** 2013-11-26

**Authors:** Weijiao Huang, Jingfeng Huang, Xiuzhen Wang, Fumin Wang, Jingjing Shi

**Affiliations:** 1 College of Southeast Land Management, Zhejiang University, Hangzhou, 310029, China; E-Mail: huangweijiao@zju.edu.cn; 2 Institute of Agricultural Remote Sensing and Information Technology, College of Environmental and Resource Sciences, Zhejiang University, Hangzhou, 310058, China; E-Mail: jjshi46@zju.edu.cn; 3 Ministry of Education Key Laboratory of Environmental Remediation and Ecological Health, Zhejiang University, Hangzhou, 310058, China; 4 Key Laboratory of Agricultural Remote Sensing and Information System of Zhejiang Province, Hangzhou, 310029, China; 5 Institute of Remote Sensing and Earth Sciences, Hangzhou Normal University, Hangzhou, 311121, China; E-Mail: wxz0516@sina.com; 6 Institute of Hydrology and Water Resources, Zhejiang University, Hangzhou, 310058, China; E-Mail: wfmwfmwfmwfm@163.com

**Keywords:** canopy reflectance, NDVI, spectral response function, sensor simulation, cross-calibration

## Abstract

Long-term monitoring of regional and global environment changes often depends on the combined use of multi-source sensor data. The most widely used vegetation index is the normalized difference vegetation index (NDVI), which is a function of the red and near-infrared (NIR) spectral bands. The reflectance and NDVI data sets derived from different satellite sensor systems will not be directly comparable due to different spectral response functions (SRF), which has been recognized as one of the most important sources of uncertainty in the multi-sensor data analysis. This study quantified the influence of SRFs on the red and NIR reflectances and NDVI derived from 31 Earth observation satellite sensors. For this purpose, spectroradiometric measurements were performed for paddy rice grown under varied nitrogen levels and at different growth stages. The rice canopy reflectances were convoluted with the spectral response functions of various satellite instruments to simulate sensor-specific reflectances in the red and NIR channels. NDVI values were then calculated using the simulated red and NIR reflectances. The results showed that as compared to the Terra MODIS, the mean relative percentage difference (RPD) ranged from −12.67% to 36.30% for the red reflectance, −8.52% to −0.23% for the NIR reflectance, and −9.32% to 3.10% for the NDVI. The mean absolute percentage difference (APD) compared to the Terra MODIS ranged from 1.28% to 36.30% for the red reflectance, 0.84% to 8.71% for the NIR reflectance, and 0.59% to 9.32% for the NDVI. The lowest APD between MODIS and the other 30 satellite sensors was observed for Landsat5 TM for the red reflectance, CBERS02B CCD for the NIR reflectance and Landsat4 TM for the NDVI. In addition, the largest APD between MODIS and the other 30 satellite sensors was observed for IKONOS for the red reflectance, AVHRR1 onboard NOAA8 for the NIR reflectance and IKONOS for the NDVI. The results also indicated that AVHRRs onboard NOAA7-17 showed higher differences than did the other sensors with respect to MODIS. A series of optimum models were presented for remote sensing data assimilation between MODIS and other sensors.

## 1. Introduction

In the past several decades, satellite remote sensing has played a vital role in providing up-to-date and detailed information for monitoring atmospheric and terrestrial environments at the regional, continental, and global scales. Such information is typically generated based on remotely sensed images processed into spectral vegetation indices [1,2]. Among the various spectral vegetation indices derived from remotely sensed imagery, one of the most widely used vegetation indices is the normalized difference vegetation index (NDVI), which is defined as the difference between the red and near-infrared (NIR) reflectance divided by their sum [3,4]. Previous studies showed that NDVI is strongly related to the fraction of absorbed photosynthetically active radiation (FPAR) [5,6], leaf area index (LAI) [7], and net primary production (NPP) [8–11]. NDVI has also been used in a range of applications including the study of vegetation–climate interactions [12–14], detection of long-term vegetation changes [15,16], assessment of vegetation functional characteristics [17–19] and modeling of the global carbon balance [10,20]. Furthermore, NDVI time series data has been successfully used in a variety of applications, including global change investigations, phenological studies, crop growth monitoring and yield prediction, drought and desertification monitoring, wildfire assessment, and climatic and biogeochemical modeling.

Since the launch of the National Oceanic and Atmospheric Administration (NOAA) satellites in 1970s, a large amount of invaluable and irreplaceable data sets have been available for global vegetation monitoring [21]. The Advanced Very High Resolution Radiometer (AVHRR) sensors onboard the NOAA satellites have provided one of the most extensive time series of remotely sensed data and continue to produce daily information regarding surface and atmospheric conditions [22]. Recently, the Moderate Resolution Imaging Spectroradiometer (MODIS) sensors onboard Terra and Aqua, designed to succeed the AVHRR instrument, has been of great importance for monitoring ecosystem variability and responses to seasonal and inter-annual environmental changes due to the improvement of both the temporal and spectral resolution relative to AVHRR. Sensors of this type, such as NOAA-AVHRR and EOS-MODIS, are appropriate for obtaining time-series data and provide more opportunities for acquiring cloud-free images by the use of composite images collected within a short period, although they are unable to avoid the influence of frequent heavy cloud cover. Many studies have demonstrated the application of these sensors to obtain large-area land-cover information [2,23–29]. Sensors of the other type, such as the Thematic Mapper/Enhanced Thematic Mapper (TM/ETM+) onboard Landsat, the High Resolution Visible/High Resolution Geometric (HRV/HRG) onboard Satellite Pour l'Observation de la Terre (SPOT), the Advanced Spaceborne Thermal Emission and Reflection Radiometer (ASTER) onboard Terra and the charge-coupled device (CCD) onboard the China Brazil Earth Resources Satellite (CBERS), have a relatively high spatial resolution, but small coverage and long revisit period. These instruments are appropriate for obtaining only detailed local information due to incomplete spatial coverage, infrequent temporal coverage with inevitable cloud contamination and the associated large data volumes or high costs that are not feasible for programs operating at a large geographical scale [2].

As a result of the ever-increasing number of Earth observation satellite systems, the user community now has access to an extensive global record of multi-sensor NDVI composites for application in biophysical monitoring and climate change modeling. Many users have found that often a combination of all available sources is more useful, as each imaging system has a different length of record as well as varied spatial, temporal, and radiometric characteristics [30]. Although the use of multi-sensor data can help to fill gaps in spatial and temporal coverage, differences between sensor characteristics can hinder the successful integration of multi-sensor datasets. Therefore, to make effective use of the long-term observation records, there has been an effort to investigate data continuity and compatibility due to drifts in calibration, filter degradation, and variations in band locations or bandwidths [31–34]. Despite these efforts, the inter-sensor VI continuity issue has remained critical and complicated. The main difficulties in the use of multi-sensor reflective spectra and NDVI time series for operational global vegetation studies arise from differences in the following: orbital overpass times [35], geometric, spectral, and radiometric calibration errors [36–41], atmospheric contamination [42,43], and directional sampling and scanning systems [44,45]. The combination of some of these factors can mitigate or exacerbate the resulting variations in solar reflective spectra [46].

In addition to the aforementioned factors, one of the most important senor characteristics, the relative spectral response function (SRF), varied among different sensors. This variation has a significant effect on the continuity of multi-sensor monitoring of global vegetation [30,47–55]. Therefore, many studies have focus on this “spectral issue”. For example, Teillet *et al*. [38] demonstrated the effects of changes in the relative spectral response on NDVI derived from AVIRIS data for a forested region in southeastern British Columbia. The results indicated that the NDVI is significantly affected by differences in the spectral bandwidth, especially for the red band, and that changes in the spatial resolution lead to less influential but more specific land cover effects on NDVI. Trishchenko *et al*. [52,53] investigated the sensitivity of the surface reflectance and NDVI to variations in the relative spectral response functions for moderate resolution satellite sensors, including various AVHRRs, MODIS, Vegetation sensor (VGT) and Global Imager (GLI). The results showed that the NDVI and reflectance were sensitive to the SRF. The significant difference in the reflectance can range from −25% to 12% for the red band and −2% to 4% for the NIR band among the “same type” AVHRR series sensors on various NOAA satellites, and even greater differences were observed for inter-comparisons of other sensor (AVHRR, MODIS, VEGETATION, and GLI). Gonsamo and Chen [46] evaluated the SRF cross-sensor comparability in the red, NIR, and SWIR reflectances, as well as the NDVI generated from large data sets representing a wide range of vegetation distributions and provided land cover independent SRF cross-sensor correction coefficients among 21 Earth observation satellite sensors. Agapiou *et al*. [56] compared the spectral sensitivity of different satellite images based on the relative spectral response function of each sensor, including ALOS, ASTER, IKONOS, Landsat 7-ETM+, Landsat 4-TM, Landsat 5-TM and SPOT 5-HRV. The results have showed that all the other sensors showed similar results and sensitivities except IKONOS. This difference for IKONOS sensor might be a result of its spectral characteristics (*i.e.*, the SRF). The results indicated that reflectances and NDVI from different satellite sensors cannot be regarded as directly equivalent.

Previous studies have typically focused on specific sensors, such as MODIS, AVHRR and TM/ETM+, whereas considerably less attention has been given to sensors such as CBERS CCD and HJ1-A/B CCD. Furthermore, few inter-calibration studies have designed for some specific crop types, e.g., paddy rice for remote sensing of crop management. Paddy rice fields make up over 11% of global cropland area [57]. Since paddy rice is grown on flooded soils (irrigated and rained), water resource management is a major concern. Seasonally flooded rice paddies have also been recognized as an important source of methane emissions, contributing over 10% of the total methane flux to the atmosphere, which may have a major impact on world climate [58,59]. Therefore, we collected rice canopy spectra from field experiments so as to address the aforementioned need and quantify the influence of the sensor spectral response function on the red and NIR reflectances and NDVI derived from 31 Earth observation satellite sensors, including CBERS CCD and HJ1-A/B CCD. For this purpose, several rice canopy spectra were obtained from two field experiments using different nitrogen levels, different species, and different transplanting dates during the rice growing season. To simulate the red and near infrared reflectances, the rice canopy spectra were convoluted with the spectral response functions of 31 Earth observation satellite sensors. NDVI values were then calculated using the simulated red and NIR reflectances. We also characterized the differences in the red reflectance, NIR reflectance and NDVI between MODIS and the other aforementioned sensors and investigated cross-sensor relationships of the NDVI as well as the red and NIR reflectances relative to MODIS. The outcomes of this study will help users to understand the differences in spectral information and provide cross-sensor relationships for detecting spatial and temporal variations in rice crops based on the use of multi-sensor data.

## 2. Materials and Methods

### 2.1. Field Experiments

The experiments were performed in 2002 and 2004 at a study site located at the Zhejiang University Experiment Farm, Hangzhou, Zhejiang Province, China (30°14′N, 120°10′E). The climate of this area is dominated by monsoon conditions with a hot summer and cool winter and with marked seasonal variations in precipitation. The average annual rainfall was 1,374.7 mm, and the average annual temperature was 17.8 °C. The soil at the study site was sandy loam paddy soil with a pH of 5.7, an organic matter content of 16.5 g·kg^−1^, and a total N content of 1.02 g·kg^−1^.

**Figure 1.**
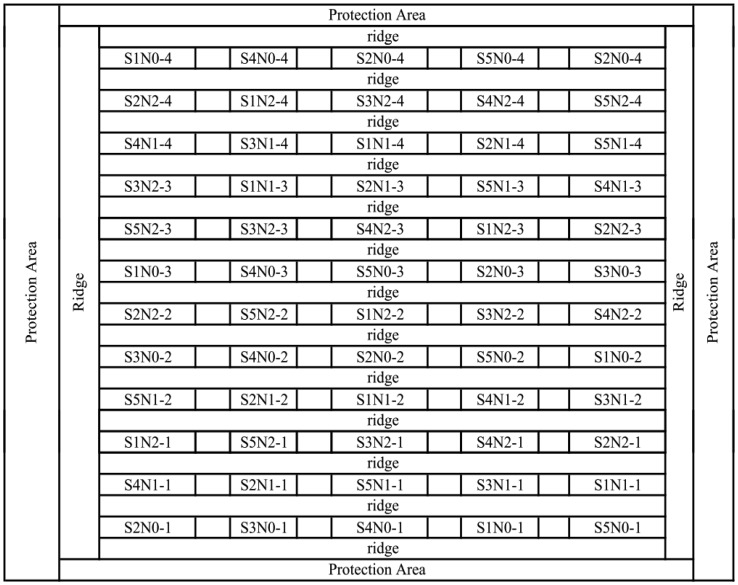
Field plot configuration of the paddy rice experiment for different nitrogen levels and species in 2002. S1, S2, S3, S4, and S5 represent Xiushui 110, Jiayu 293, Jiazao 312, Z00324, and Xieyou 9308, respectively. N0, N1, and N2 represent 0 kg·ha^−1^, 140 kg·ha^−1^, and 240 kg·ha^−1^ of pure nitrogen, respectively.

In 2002, the experiment field comprised 60 plots in which different rice cultivars (xiushui 110, Jiayu 293, Jiazao 312, Z00324 and Xieyou 9308) were sown on 2 June in a completely randomized block design with four replicates (Figure 1). The Xieyou 9308 cultivar was a hybrid rice, whereas the others were common rice. Jiayu 293, Jiazao 312, Z00324 and Xieyou 9308 were indicia rice, and Xiushui 110 was japomica rice. The three different levels of N fertilization including no fertilizer (0 kg·ha^−1^), a normal application rate (120 kg·ha^−1^), and a superabundant dose of urea (240 kg·ha^−1^)were applied in the proportions of 45% base fertilizer, 35% tillering fertilizer, and 20% heading fertilizer for each cultivar. In addition, 533.3 kg of Ca(H_2_PO_4_)_2_ ha^−1^ was applied as a base fertilizer with 300 kg of KCl ha^−1^ as a heading fertilizer.

**Figure 2.**
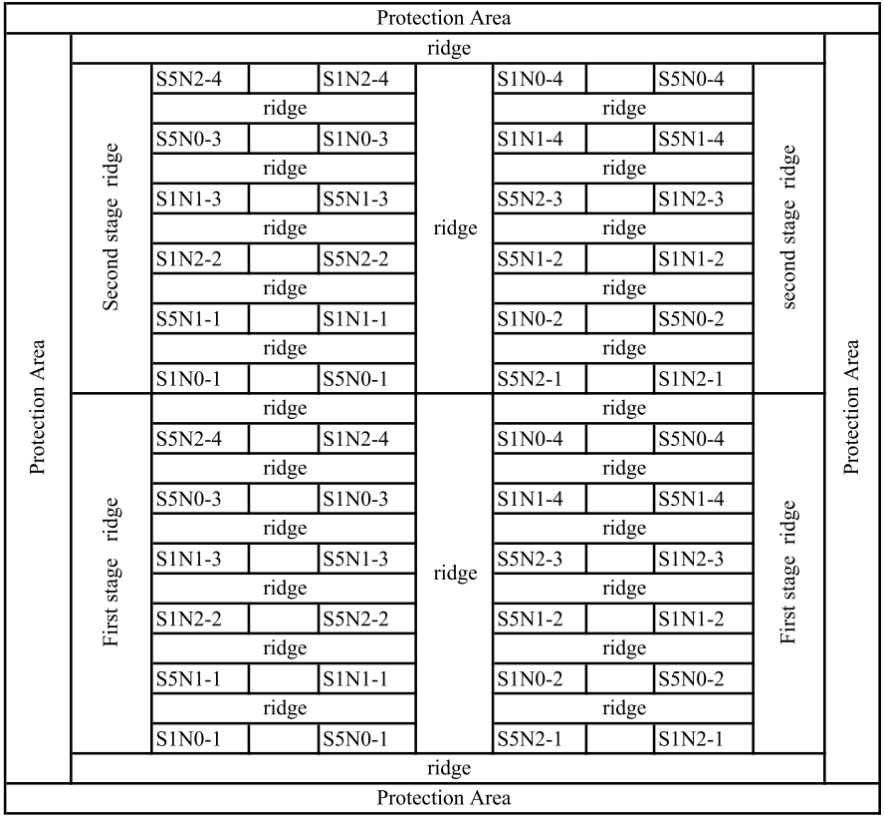
Field plot configuration of the paddy rice experiment for different nitrogen levels, species and transplanting dates in 2004. S1, S2, S3, S4, and S5 represent Xiushui 110, Jiayu 293, Jiazao 312, Z00324, and Xieyou 9308, respectively. N0, N1, and N2 represent 0 kg·ha^−1^, 140 kg·ha^−1^, and 240 kg·ha^−1^ of pure nitrogen, respectively.

In 2004, the experiment field was consisted of 48 plots of size 4.6 m × 5.46 m. One half of the plots were used for the first experiment and the remaining plots were used for the second experiment (Figure 2). Each experiment involved four replicates of two rice cultivars (xiushui 110 and Xieyou 9308), and the plant density was 45 plants m^−2^. The first experiment was seeded on 30 May 2004 and the second experiment was seeded on 15 June 2004. Both seedling sets were transplanted to the field one month later. Nitrogen levels and all treatments were identical to the 2002 experiment.

### 2.2. Canopy Reflectance Measurement

Canopy reflectance measurements were performed under clear-sky conditions at approximately midday (10:00–14:00 LST) using an Analytical Spectral Devices Full Range Spectroradiometer (FieldSpec-FR, ASD, Boulder, CO, USA) during the growing stage, including the early tillering stage, peak tillering stage, gestation stage, heading stage, milky stage and ripening stage. The spectral range and the field of view of the sensor were 350–2,500 nm and 25°, respectively. The sampling interval over the 350–1,000 nm range is 1.4 nm with a resolution of 3 nm, and over the 1,000–2,500 nm range the sampling interval is about 2 nm and the spectral resolution is 10 nm. Fieldspec can acquire both radiance and reflectance data. The reflectance factor of the target is automatically calculated by the instrument as the ratio between the incident radiation, reflected from the surface target, and the incident radiation reflected by a BaSO_4_ white reference, which is regarded as a Lambertian reflector (the reflectance factor will be called ‘reflectance’ throughout the whole present manuscript. Reflectance spectra were collected from a distance of 1.0 m vertically above the canopies with a field of view of 25°. A single reflectance measurement was obtained as the average of 10 scans to minimize instrumental noise.

**Figure 3.**
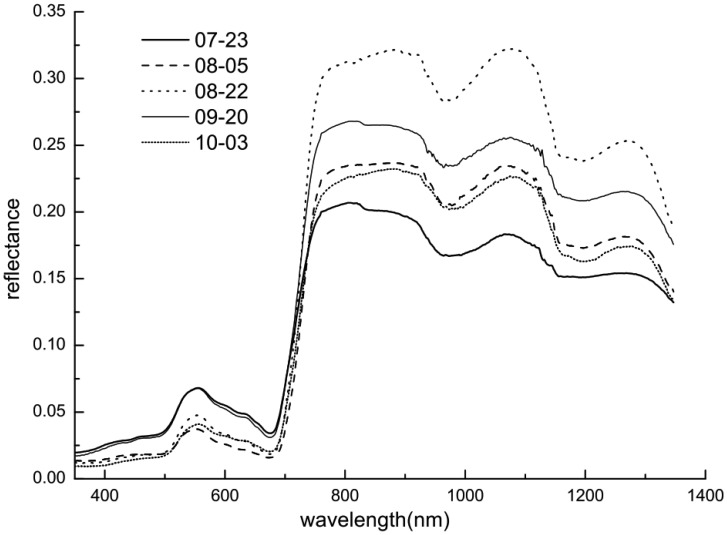
Average reflectances of the rice canopy at the N1 level of N fertilization at different growth stages in 2002.

In 2002 spectra were collected on eight dates at distinctive growth stages (17, 23, 30 July, 5, 22, 31 August, 20 September and 3 October) for all experimental field plots. In 2004 measurements were acquired using the same scheme described above on six dates (20 July, 8, 28 August, 22 September and 5, 24 October) during the rice growth stages. For example, the spectral reflectances of the rice canopy at the N1 level of N fertilization at different growth stages in 2002 are shown in Figure 3. As expected, the reflectance spectra of the canopy were significantly different among the various growth stages.

### 2.3. Satellite Sensors and Their Relative Spectral Response Functions

This study included the evaluation of both high and very high spatial resolution multispectral satellite sensors. Satellite images acquired from these sensors are often used for the detection of vegetation dynamics. Table 1 shows the spectral and spatial characteristics of the satellites sensors used in this study.

The spectral characteristics of the sensor could be characterized by the relative spectral response function (SRF) for each band, which is defined as the ratio of the output signal to the incident flux as a function of wavelength, normalized to the peak value of unity [60]. The SRF indicates the relative sensitivity of a sensor to incoming energy at each wavelength. The relative spectral response function can be described by three key factors, *i.e.*, the center wavelength (CWL), the full width at half the maximum (FWHM), and the shape. The entire set of band SRFs determines the spectral performance of the radiance data. The FWHM, also called bandwidth, indicates the spectral resolution capability of the detector. The SRF shape varies depending on the manner that the sensor disperses and detects the incident light. Sensor models characterize the process converting the spectral response of the land surface-atmosphere system into digital numbers, and the SRF is one of the most important components of sensor modeling system [61].

**Figure 4.**
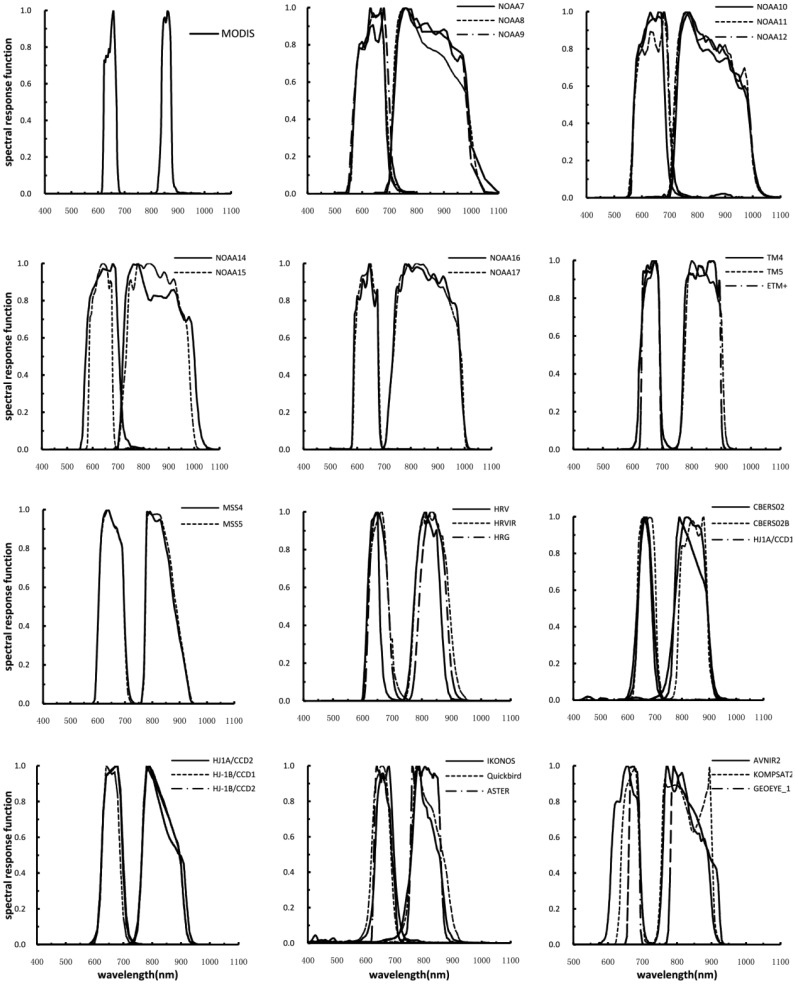
Relative spectral response functions of the red and NIR channels for the sensor systems simulated in this study.

The relative spectral response functions of the red and NIR channels for the different sensors used in this study are shown in Figure 4 and Table 1. These sensors include NOAA7-17AVHRRs, SPOT HRV, Landsat TMs, ETM+, MSS, HJ1A/B CCDs, CBERS CCDs, IKONOS, QuickBird, and ALOS AVNIR2. The SRFs were obtained from the operator's website or by personal communication. Though similar, these curves differ in their shape, central wavelength location, bandwidth, and degree of overlap between channels, especially with respect to the transition from the chlorophyll absorption band to the foliage reflection band (0.68–0.72 μm). The spectral response functions of the sensors vary from one another, especially in the NIR region.

A comparison of the spectrally matched bands between MODIS and other sensors indicated that the spectral ranges of the MODIS red and NIR channels are much narrower than those for the other sensors and have no overlap with each other over the vegetation transition band (Figure 4). It was clearly evident that the gap between the red and NIR band of MODIS was wider than the gap between the red and NIR band of the other sensors, even where an overlap exists. Thus, the other sensor bands are closer to the red edge relative to MODIS. It was noted that the Landsat TMs, SPOT HRVs and NOAA AVHRRs response curves varied depending on the version of the instrument that was employed. The bandwidths of the Landsat7 ETM+ channels are narrower than those of TM on Landsat4 and 5. A similar result was also found for the new instrument AVHRR3 onboard NOAA15, 16 and 17. The channels of the new AVHRR3 instrument have narrower bandwidths and a much smaller overlap over the vegetation transition band than do the other AVHRRs. This indicated that a direct comparison of spectral reflectance or vegetation indices produced by various sensors should be performed with caution.

### 2.4. Bandpass Target Reflectance Simulation

Some sensor simulation methods take into account both the spectral and spatial differences of the sensor [62]. In this study, our objective was to assess the spectral band differences solely caused by different spectral response functions without considering atmospheric intervention. Moreover, the only variation in the simulation based on the same spectra was the spectral response function of the sensor, and this is the only parameter that enables us to compare the different sensors.

To simulate the multiple bands from hyperspectral bands, the reflectance of the narrow hyperspectral bands must be convoluted with the spectral response function of each band that simulates the bands of the satellite sensors. Prior to simulation, each hyperspectral central wavelength was linked with the mean SRF value (in the range of the full width half maximum (FWHM) of the hyperspectral band) of the simulated band. This approach was similar to the method proposed by Franke *et al*. [63]. The equation for simulating the reflectance of the sensor can be expressed as follows:

(1) $$\bar{\rho }\left(\lambda \right)=\frac{\int_{\lambda_{2}}^{\lambda_{1}}\rho \left(\lambda \right)\text{R}\left(\lambda \right)\text{d}\lambda }{\int_{\lambda_{2}}^{\lambda_{1}}\text{R}\left(\lambda \right)\text{d}\lambda }$$

where ρ̄(λ) is the simulated reflectance value for a given sensor, ρ(λ) is the target reflectance observed at a specific wavelength λ, R(λ)is the spectral response function value at a specific wavelength λ. The spectral response function was integrated with the target reflectance to generate the band pass value. A set of paddy rice spectra that encompassed a range of variability in the surface reflectance was used in this computation. The band average values for the red and NIR bands were computed using Equation (1).

The normalized difference vegetation index was then calculated as follows:

(2) $$\text{NDVI}=\frac{{\bar{\rho }}_{\text{NIR}}‐{\bar{\rho }}_{\text{red}}}{{\bar{\rho }}_{\text{NIR}}+{\bar{\rho }}_{\text{red}}}$$

where ρ̄_red_ and ρ̄_NIR_ are the bandpass reflectances in the red and NIR channels, respectively. A comparison between the band average reflectance for various targets was performed to quantify the effect of different SRFs in various sensors.

### 2.5. Comparison of Reflectance and NDVI between MODIS and Other Sensors

A comparative analysis of the red/near-infrared reflectance and NDVI for various sensors was performed using two statistical measurements namely, the relative percentage difference (RPD) and absolute percentage difference (APD). The computations were conducted for different sensors with the MODIS sensor as a reference, and the following formula was used to compute the RPD and APD:

(3) $$\text{RPD}=\frac{\rho_{i}-\rho_{\text{MODIS}}}{\rho_{\text{MODIS}}}\times 100\%$$

(4) $$\text{APD}=\left|\frac{\rho_{i}-\rho_{\text{MODIS}}}{\rho_{\text{MODIS}}}\right|\times 100\%$$

However, it was not clear whether the differences in the reflectance and NDVI between MODIS and the other sensors were statistically significant. Thus, a paired Student t test was used to compare the differences in the reflectance and NDVI between MODIS and the other sensors in this study.

## 3. Results and Discussion

The simulated reflectances for the 31 different satellite sensors were calculated to quantify the influence of the varying spectral response functions on the target reflectance in the red band, NIR band and NDVI. The effects of the SRF on the reflectance in the red and NIR channels and NDVI were then evaluated based on RPD and APD with respect to the MODIS values.

### 3.1. The Effect of the SRF on the Reflectance in the Red Channel

The minimum, maximum and mean values as well as the standard deviation of the red reflectance for various satellite sensors simulated using paddy rice canopy reflectance data are listed in Table 2. For Terra-MODIS, the simulated reflectance in the red channel ranged from 0.0128 to 0.1337, and the mean reflectance value was 0.0354. In this study, we used this instrument as a reference due to its high spatial and temporal coverage. The largest mean reflectance in the red was found for IKONOS at 0.0460, followed by the AVHRR2 onboard NOAA12 (0.0454), NOAA14 (0.0453) and NOAA11 (0.0441). The lowest mean reflectance was observed for GEOEYE-1 at 0.0313, followed by Landsat7-ETM+ (0.0336), KOMPAST2 (0.0343) and MODIS (0.0354).

The RPDs for all other sensors in this study in the red band with respect to MODIS were between −21.02% and 77.73% (Table 2). The mean RPD between MODIS and various other sensors, averaged over 447 samples, ranged from −12.67% (GEOEYE-1) to 36.30% (IKONOS). Similar to GEOEYE-1, Landsat7 ETM+ and KOMPSAT2 showed negative RPDs of −5.6% and −3.58%, respectively. The comparison indicated that the red reflectances of GEOEYE-1, Landsat7 ETM+ and KOMPSAT2 were smaller than that of MODIS. With the exception of the above three sensors, the red reflectances of the other sensors were larger than that of MODIS. It was observed that AVHRR2 onboard NOAA7, 9, 11, 14, and 12 (20.66%, 27.96%, 29.04%, 32.98%, 33.84%, respectively) had a higher mean RPD with respect to MODIS than did AVHRR1 and AVHRR3 onboard NOAA8 and 10 (18.11% and 17.10%, respectively) and NOAA15, 16, 17(10.93%, 9.07%, and 5.99%, respectively). The mean RPD for Landsat4 TM, Landsat5 TM, SPOT1 HRV, SPOT4 HRVIR and SPOT5 HTG were 1.11%, 0.95%, 6.10%, 14.13% and 3.75%, respectively. The mean RPD for CCD1 onboard HJ1-A and HJ1-B were 6.27% and 2.81%, respectively, whereas the values for CCD2 onboard HJ1-A and HJ1-B were 13.25% and 11.27%, respectively. The ASTER and ALOS AVNIR2 sensors having a high spatial resolution showed small RPDs of 3.91% and 4.25%, respectively, as compared to QuickBird and IKONOS.

The minimum APD between MODIS and the other sensors was observed for Landsat5 TM with a value of 0.0013%, and the maximum APD was observed for IKONOS with a value of 77.73%. The smaller difference was identified for Landsat5-TM with a mean APD value of 1.28%, followed by Landsat4-TM (1.36%), HJ-1B(CCD1) (2.85%) and KOMPSAT2(3.62%). The largest difference was observed for IKONOS with a mean APD value of 36.30%, followed by AVHRR2 onboard NOAA12 (33.84%), 14 (32.98%) and 11 (29.04%). AVHRR2 onboard NOAA7, 9, 11, 14, and 12 had higher mean APD with respect to MODIS than did the AVHRR1 and AVHRR3 onboard NOAA8, 10 and NOAA15, 16, 17. With the exception of Landsat5 TM, Landsat4 TM, HJ-1B CCD1, KOMPSAT2, Terra ASTER and Landsat7 ETM+, the mean APD value of the other sensors were equivalent to the RPD with respect to MODIS (Table 2).

**Figure 5.**
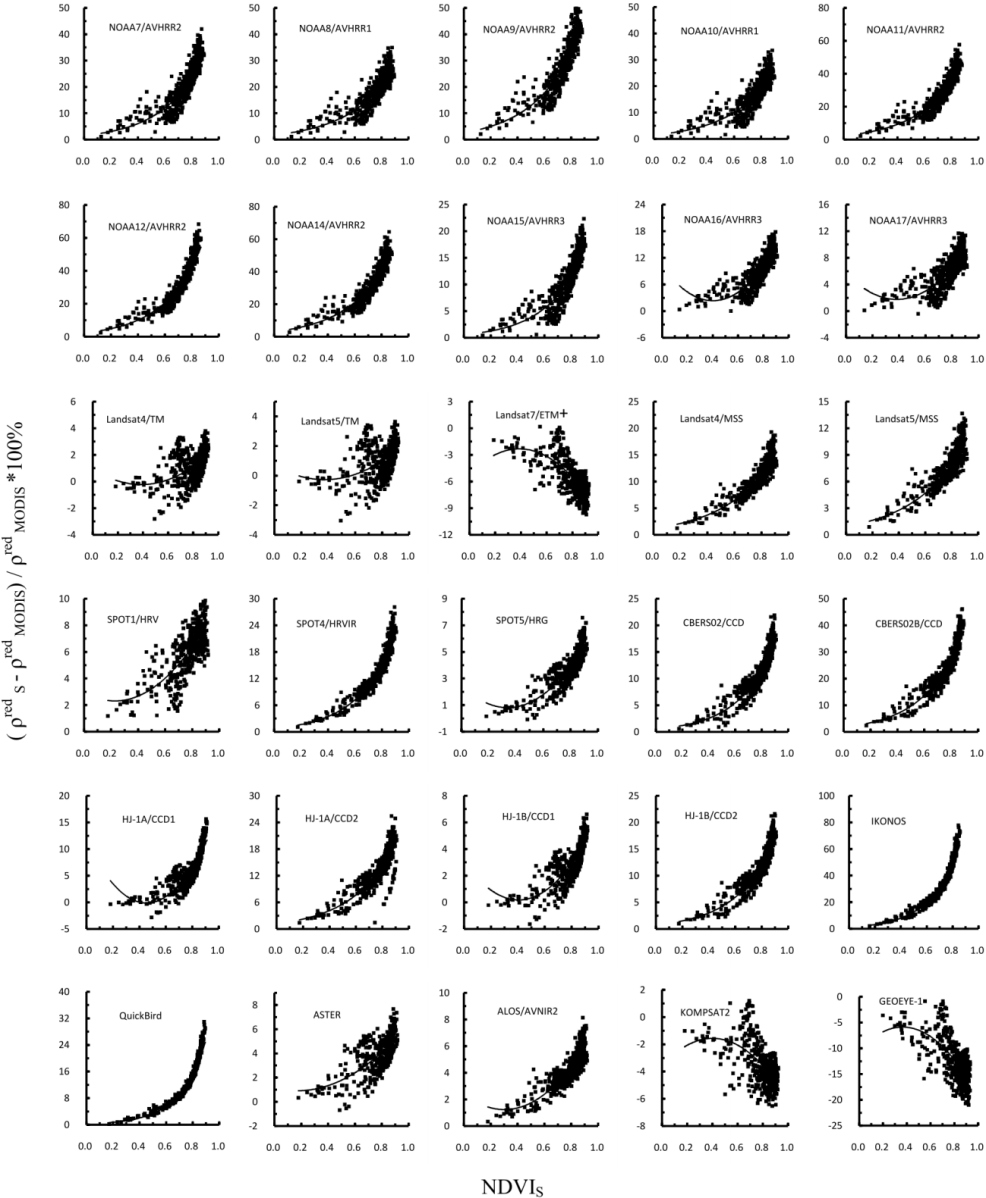
Relative percentage differences in the red channel reflectances with respect to Terra MODIS. All data points are plotted versus the NDVI of a particular sensor. Parameters of the fitting curves are given in Table 3.

Further analysis of the significance based on paired t tests indicated that the differences in the red reflectance between MODIS and the other sensors were highly significant (*p* < 0.0001). The analysis of difference indicated that it is important to apply the spectral correction when using combined data from MODIS and the other sensor. The discrepancies caused by different SRFs may be corrected using the second-degree polynomial functions and exponential functions, as shown in Figure 5. The curves were produced by fitting the data points. Figure 5 shows that the RPD in the red reflectance for all other sensors in this study increased with increasing target NDVI values, excluding KOMPSAT2, Landsat7 ETM+ and GEOEYE-1. The value of the mean absolute difference for KOMPSAT2, Landsat7 ETM+ and GEOEYE-1 was negative. Thus, the direction of the curves for these three sensors was opposite that of the other sensors. The coefficients of the quadratic and exponential functions that best fit the data and correlation coefficient of the fit for each sensor for the red reflectance are shown in Table 3. The quality of the fit was high for AVHRRs, IKONOS and CBERS02B CCD, whereas data for the TM and ASTER sensors were more scattered.

### 3.2. The Effect of the SRF on the Reflectance in the NIR Channel

The descriptive statistics of the NIR reflectances for various satellite sensors simulated using paddy rice canopy reflectance are listed in Table 4. For Terra MODIS, the simulated reflectance in the NIR channel ranged from 0.0927 to 0.5023, and the mean reflectance value was 0.3019. The highest mean reflectance value in the NIR channel was 0.3019 for MODIS, followed by SPOT5 HRG (0.3008), CBERS02B CCD (0.3006) and GEOEYE-1 (0.3005). The lowest mean reflectance was observed for NOAA8 AVHRR1 with a value of 0.2757, followed by IKONOS (0.2782), AVHRR2 onboard NOAA-11 (0.2784) and NOAA-9 (0.2787).

Among the studied sensors, the minimum RPD with respect to MODIS in the NIR band was −12.29% for NOAA14 AVHRR2, and the maximum RPDs was 56.94% for SPOT5 HRG. The mean RPD of the NIR reflectance varied from −8.52% (NOAA8 AVHRR1) to −0.23% (SPOT5 HRG). The mean RPD for all of the sensors with respect to MODIS was negative. The mean RPD was within −7.66% to −6.93%, −5.15% to −4.98%, −1.20% to −1.06% and −0.47% to −0.40% for the AVHRR2s, AVHRR3s, HJ-1A/B CCDs and Landsat TMs sensors, respectively. The mean RPDs for SPOT1 HRV, SPOT4 HRVIR and SPOT5 HRG were −0.99%, −0.57% and −0.23%, respectively.

The largest mean APD in the NIR channel was observed for NOAA8 AVHRR1 with a value of 8.71%, followed by AVHRR2 onboard NOAA11 (7.86%), NOAA9 (7.76%) and IKONOS (7.69%). For the AVHRR2 instruments, the mean APDs ranged from 7.14% to 7.86%. AVHRR3 had a little smaller APD than did AVHRR1s and AVHRR2s at 5.19%, 5.30% and 5.36% for NOAA17, NOAA15 and NOAA16, respectively. The smallest difference was identified for CBERS02B CCD with the mean APD value of 0.84%, followed by GEOEYE-1 (0.91%), SPOT5 HRG (0.94%) and Landsat4-TM (0.98%). The mean APD for Landsat TMs, MSSs, and SPOT4 HRVIR ranged from 0.98% to 1.20%, whereas the values ranged from 1.62% to 1.70% for the HJ CCDs.

**Figure 6.**
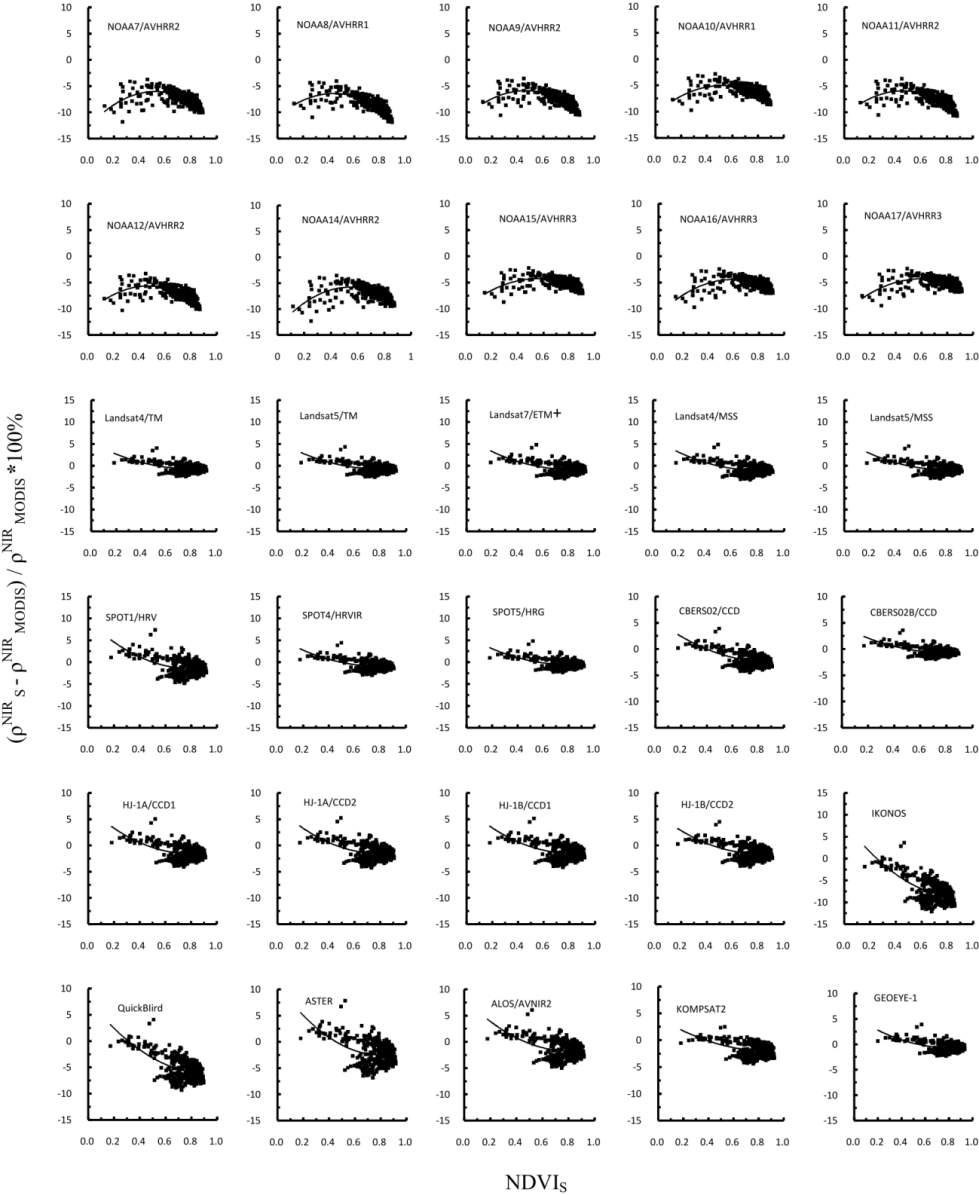
Relative percentage differences in the NIR channel reflectances with respect to Terra MODIS. All data points are plotted versus the NDVI of a particular sensor. Parameters for the fitting curves are given in Table 5.

The analysis using paired t tests indicated that the differences in the NIR reflectance between MODIS and other sensors were significant at the *p* < 0.0001 level. The discrepancies caused by different SRFs may be corrected using second-degree polynomial functions, as shown in Figure 6 and Table 5.

These curves were produced by fitting the data points. The RPDs for AVHRRs onboard NOAA7-17 showed two distinct trends with increasing target NDVI values. Specifically, the difference increased as the target NDVI value increased from 0 to 0.5; however, as the target sensor NDVI value increased above 0.5, the RPD began to decrease. The observed trends in the NIR reflectance differences are likely due to the inclusion of either the red edge or leaf liquid water absorption regions in the broad bandpass of AVHRR channel 2. The coefficients of the quadratic functions that best fit the data and correlation coefficient of the fit for each sensor for NIR reflectance were given in Table 5. The quality of the fits was higher for AVHRRs, IKONOS and QuickBird than other sensors.

### 3.3. The Effect of the SRF on the NDVI

Table 6 showed the minimum, maximum and mean values as well as the standard deviation of the NDVI for various satellite sensors. For Terra MODIS, the NDVI ranged from 0.177 to 0.9237, and the mean NDVI value was 0.7756. The lowest mean NDVI was observed for KONOS at 0.7043, followed by the AVHRR2 onboard NOAA12 (0.7071), 14 (0.7081), and 11 (0.7131). The largest mean reflectance was observed for GEOEYE-1 at 0.7973, followed by Landsat7 ETM+ (0.7845), KOMPAST2 (0.7788) and MODIS (0.7756).

Table 6 showed that the RPDs in the NDVI for all studied sensors range from −35.24% to 14.16%. IKONOS showed the lowest mean RPD of −9.32% with respect to MODIS. Similar to IKONOS, AVHRR2 onboard NOAA12, 14, 11, 9 and 7 had RPDs of −9.29%, −9.29%, −8.56%, −8.37%, and −6.72%, respectively. However, AVHRR1 onboard NOAA8 and 10 and AVHRR3 onboard NOAA15, 16, and 17 showed slightly higher mean RPDs of −6.56%, −5.61%, −3.85%, −3.57%, and −2.87%, respectively. The largest mean RPD was observed for GEOEYE-1 at 3.10%, followed by Landsat7 ETM+ (1.28%), KOMPSAT2 (0.48%), Landsat5 TM (−0.18%) and Landsat4 TM (−0.21%). The mean RPDs for SPOT1 HRV, SPOT4 HRVIR and SPOT5 HTG were −1.55%, −3.0% and 0.77%, respectively. The mean RPD for CCD1 onboard HJ1-A and HJ1-B was −1.37% and −0.72%, respectively, whereas the values for CCD2 onboard HJ1-A and HJ1-B were −3.07% and −2.62%, respectively. The sensors having a high spatial resolution, such as ASTER and ALOS AVNIR2, showed larger RPDs of −1.24% and −1.16%, respectively.

The minimum mean APD in the NDVI between MODIS and the other sensors was observed for Landsat4 TM with a value of 0.59%, followed by Landsat5 TM (0.60%), KOMPSAT2(0.75%) and SPOT HRG (0.92%). The mean APDs were 1.27%, 1.34%, 1.43%, 1.59%, 1.62% and 1.82% for ALOS AVNIR2, LANDSAT7 ETM+, Terra ASTER, HJ-1A CCD, SPOT1 HRV and LANDSAT5 MSS, respectively. Compared to Landsat5 MSS and HJ-1A CCD1, the mean APD was 2.47%, 2.64% and 3.09% for LANDSAT4 MSS, HJ-1B CCD2 and HJ-1A CCD2, respectively. The maximum mean APD between MODIS and the other sensors was observed for IKONOS with a value of 9.32%, followed by AVHRR2 onboard NOAA12 (9.29%), NOAA 14 (9.29%) and NOAA 11 (8.56%). AVHRR2 onboard NOAA7, 9, 11, 14, and 12 showed higher mean APDs with respect to MODIS than did the AVHRR1 and AVHRR3 onboard NOAA8 and 10 (6.57% and 5.62%) and NOAA15, 16, 17 (3.87%, 3.59%, and 2.89%).

**Figure 7.**
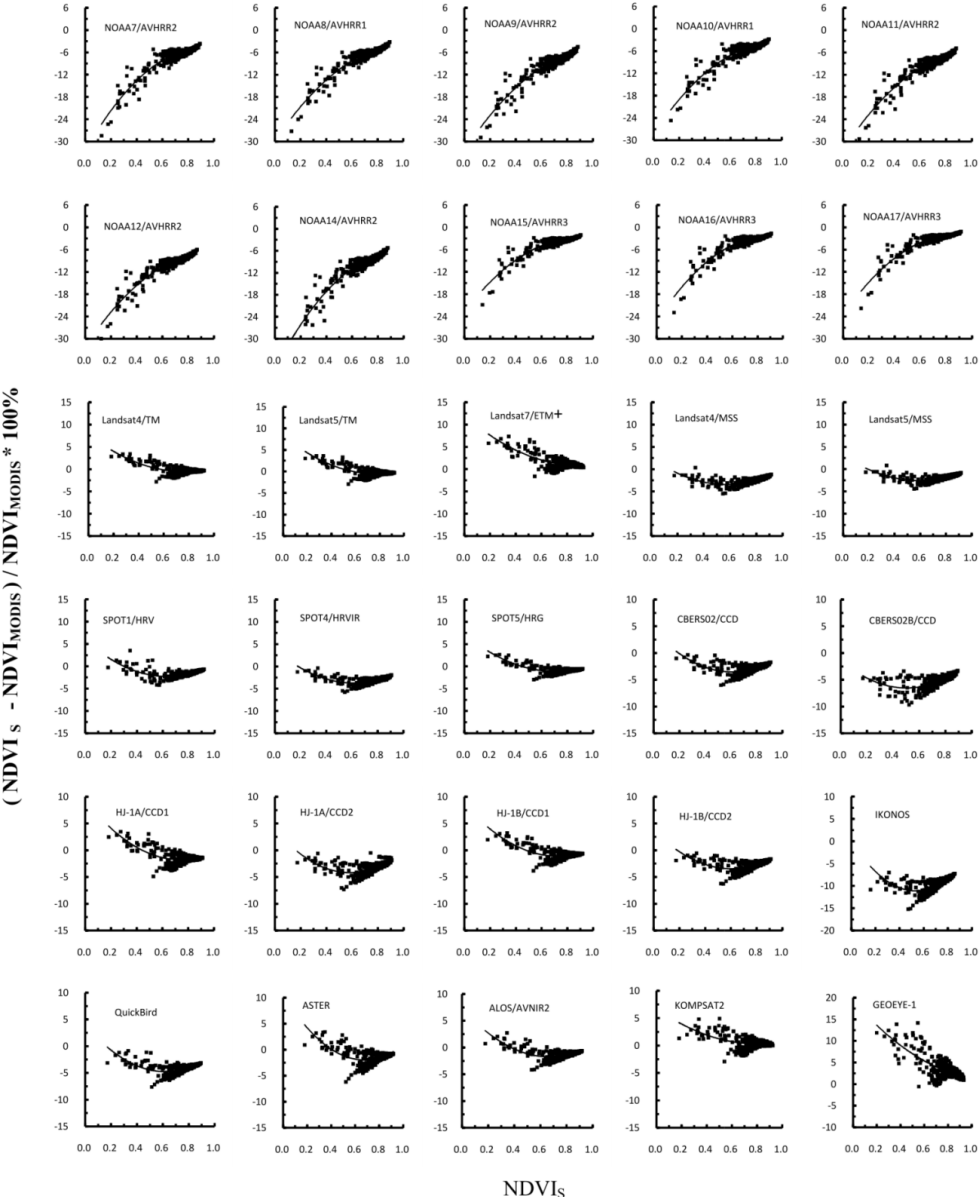
Relative percentage differences in NDVI with respect to Terra MODIS. All data points are plotted versus NDVI of particular sensor. Parameters of fitting curves are given in Table 7.

The paired t tests confirmed that there were significant differences (*p* < 0.0001) in NDVI between MODIS and the other sensors. The discrepancies caused by different SRFs may be corrected using second-degree polynomial functions, as shown in Figure 7. The curves were produced by fitting the data points. It was observed that the RPDs in the NDVI with respect to MODIS for AVHRRs increased with increasing target NDVI values. The coefficients of the quadratic functions that best fit the data, as well as the correlation coefficient of the fit for each sensor for the NDVI, are provided in Table 7. The quality of the fit was high for the AVHRRs, whereas the data for the KOMPSAT2 sensors were more scattered.

**Figure 8.**
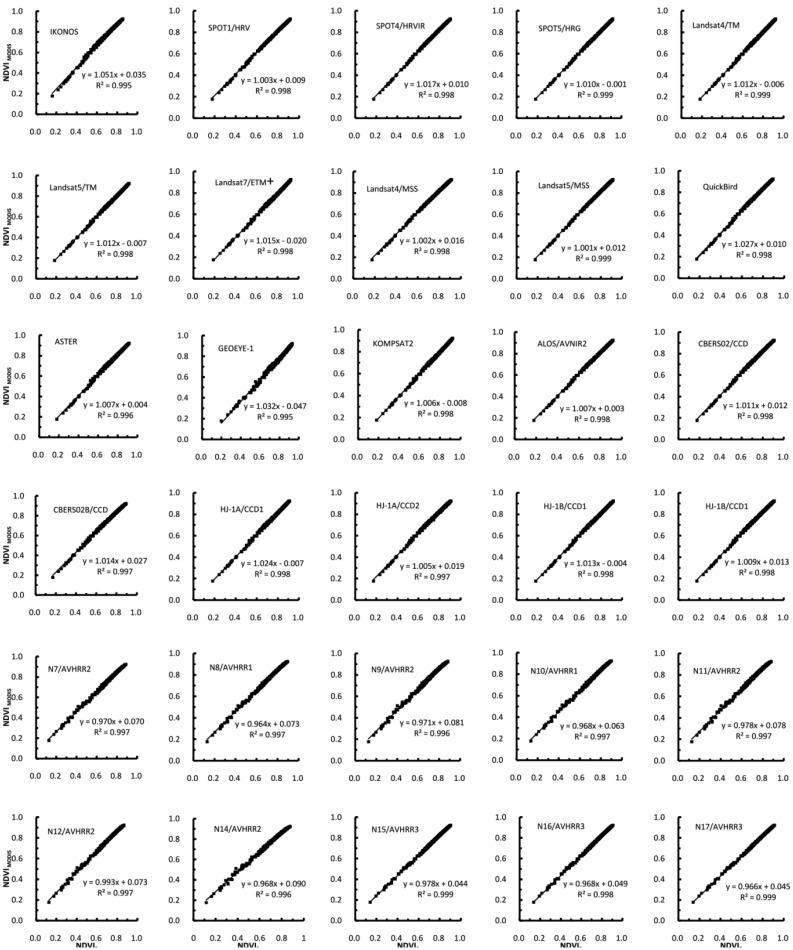
Interrelationships between the NDVIs from selected pairs of sensors.

A comparison of the NDVIs simulated for different sensors showed that the relationship between MODIS and other the instrument NDVI values can be evaluated using a linear regression model (Figure 8). The linear regression relationships between the NDVI values of the various sensor combinations presented in Figure 8 were all significant at *p* < 0.0001. Additionally, the coefficient of determination was higher than 0.99 in almost every case. However, the relationships were not 1:1. The intercept values for all sensors ranged from −0.047 (GEOEYE-1) to 0.090 (NOAA14). The slope values varied from 0.965 (NOAA8) to 1.051 (IKONOS), and the r^2^ values varied from 0.995 (IKONOS) to 0.999 (SPOT5/HRV). The results showed substantial differences between the sensor systems, which if uncorrected, would significantly bias the estimates of any biophysical parameters derived from these sensors. Even for a nominally continuous system, the NDVIs from the Landsat-ETM+ sensor differed from the values from the earlier TM sensor; therefore, the relationship between MODIS and each sensor varies. The results indicated that the relationships between the NDVIs were sufficiently robust in all cases to allow for corrections between one system relative to MODIS.

### 3.4. Comparison with Previous Studies

The results of the current study were consistent with the previous research by Trishchenko *et al.* [53]. The study investigated the effect of the SRF on reflectance and NDVI of the moderate resolution satellite sensors, including MODIS and AVHRRs. They found differences in the red band reflectance between MODIS and other sensors were relatively higher than that in the NIR band reflectance. This phenomenon of higher relative red band differences was observed in this study. In addition, the findings of comparison of MODIS and AVHRR NDVI data included in our study were identical with other studies. The MODIS NDVI data were found to be greater than the AVHRR NDVI data in an analysis over Senegal [64], South Florida [65], the Southern Great Plains of the United States [53] and simulation of MODIS and AVHRR data [48].

Our results also agreed well with the findings of other studies [30,46,48] related to the NDVI cross-sensor correction. The simulations by Steven *et al.* [48] were based on measurements of a full range of sugar beet and maize canopies. Whereas the study by van and Gonsamo *et al.* [30,46] included large sets of measured backgrounds as input to radiative transfer models (*i.e.*, SAIL, PROSPECT and 6S) to simulate canopy spectra for a large range of possible global vegetation conditions. They used simple linear regression models for cross-sensor correction. The slope and intercept values of the linear regression relationships between the NDVI values of the various sensor combinations for SPOT, MSS, TM, ETM+, IKONOS and QuickBird with an MODIS reference were almost identical to those in the present study.

This study indicated that reflectance and NDVI derived from different sensors cannot be considered as directly equivalent and NDVI based on surface reflectance can be corrected for spectral band effects. Our study was based on simulating the spectral band responses of different satellite sensors from field reflectance data. The advantages of this method are that the results are not affected to any extent by calibration errors or atmospheric interactions and the simulations can be performed for any combination of sensors for direct comparisons. However, it should be noted that other instrumental effects, such as spatial sampling and the radiometric resolution, and atmospheric corrections are not accounted for by this approach. Miura concluded that the MODIS versus AVHRR3 and AVHRR2 cross-sensor NDVI relationships were subject to larger difference due to the NIR bands which showed differential sensitivities to the atmospheric water vapor effects between sensors [66]. Therefore, accurate calibrations and atmospheric corrections are also essential for combination of data from different satellite systems.

## 4. Conclusions

Long-term monitoring of the Earth's environment using multi-source satellite sensors requires consistent and comparable measurements. However, the combined use of the multi-source satellite data sets requires a detailed evaluation of their compatibility and consistency to avoid artifacts. In this study, rice canopy spectra were collected from field experiments so as to quantify the effect of the sensor spectral response function on the red and NIR reflectances and NDVI derived from 31 Earth observation satellite sensors, including CBERS CCD and HJ1-A/B CCD. This study not only included sensors having a coarse spatial resolution, such as AVHRRs from NOAA-7 to NOAA-17 and MODIS, but also included sensors having a medium spatial resolution, such as Landsat TMs and MSSs, SPOT HRV, HRVIR and HRG, CBERS CCDs, and HJ CCDs. Moreover, we also showed sensors having a relatively high spatial resolution, such as IKONOS, QuickBird, KOMPSAT2 and GEOEYE-1. All of the sensors were compared to the Terra MODIS, which was chosen as a reference. Based on field reflectance data, we simulated the spectral band responses of different satellite sensors.

The results showed that the mean RPD in the red reflectance for all other sensors with respect to MODIS ranged from −12.67% (GEOEYE-1) to 36.30% (IKONOS). For GEOEYE-1, Landsat7 ETM+ and KOMPSAT2, the negative RPD indicated that red reflectance of MODIS was larger than that of these sensors. The mean APD in red reflectance varied from 1.28% (Landsat5-TM) to 36.30% (IKONOS). AVHRR2 onboard NOAA7, 9, 11, 14, and 12 showed a higher mean APD with respect to MODIS than the AVHRR1 and AVHRR3 onboard NOAA8 and 10 (18.11% and 17.10) and NOAA15, 16, and 17(10.93%, 9.07%, and 5.99%).

The mean RPD in the NIR reflectance for all other sensors with respect to MODIS ranged from −8.52% (NOAA8 AVHRR1) to −0.23% (SPOT5 HRG). It was observed that the mean RPD for all sensors with respect to MODIS was negative. The mean RPD ranged from −7.66% to −6.93%, −5.15% to −4.98%, −1.20% to −1.06% and −0.47% to −0.40% for the AVHRR2s, AVHRR3s, HJ CCDs and Landsat TMs, respectively. The largest mean APD was observed for NOAA8 AVHRR1 at 8.71%, and the smallest difference was observed for CBERS02B CCD at 0.84%. For the AVHRR2 instruments, the mean APDs were within the range of 7.14% to 7.86%. The AVHRR3 showed slightly smaller APD than did AVHRR1s and AVHRR2s, which were 5.19%, 5.30% and 5.36% for NOAA17, 15, and 16, respectively. The mean APD for the Landsat TMs, MSSs, and SPOT4 HRVIR was 0.98% to 1.20% and was 1.62% to 1.70% for the HJ CCDs.

With respect to MODIS, the mean RPDs in the NDVI for all sensors in this study ranged from −9.32% to 3.10%. The mean APDs in the NDVI varied from 0.59% (Landsat5-TM) to 9.32% (IKONOS). The mean APD was 1.27%, 1.34%, 1.43%, 1.59%, 1.62% and 1.82% for ALOS AVNIR2, Landsat7 ETM+, Terra ASTER, HJ-1A CCD, SPOT1 HRV and LANDSAT5 MSS, respectively. Comparison with Landsat5 MSS and HJ-1A CCD1, the mean APD was 2.47%, 2.64%, 3.09% for LANDSAT5 MSS, HJ-1B CCD2 and HJ-1A CCD2. AVHRR2 onboard NOAA7, 9, 11, 14, 12 has higher mean APD with respective to MODIS than the AVHRR1 and AVHRR3 onboard NOAA8, 10 (6.57%, 5.62%) and NOAA15, 16, and 17 (3.87%, 3.59%, and 2.89%).

The results of paired t tests showed that there were significant differences (*p* < 0.0001) between MODIS and all the other sensors regarding the red reflectance, NIR reflectance and NDVI. A series of optimum models were provided for spectral corrections between MODIS and other sensors. This result offers improved opportunities for monitoring crops through the growing season and the prospects of better continuity of long-term monitoring of vegetation responses to environmental change.

## Figures and Tables

**Table 1.** Characteristics of satellite sensors used in this study.

**Satellite Sensor**	**Launch Date**	**Revisit Time (Days)**	**Spectral Range (μm)**		**Spatial Resolution GSD at Nadir (Meters)**

			**Red**	**Near IR**	
NOAA7 AVHRR2	1981/6/23	1	0.58–0.68	0.725–1.00	1,100
NOAA8 AVHRR1	1983/3/28	1	0.58–0.68	0.725–1.10	1,100
NOAA9 AVHRR2	1984/12/12	1	0.58–0.68	0.725–1.00	1,100
NOAA10 AVHRR1	1986/9/17	1	0.58–0.68	0.725–1.10	1,100
NOAA11 AVHRR2	1988/9/24	1	0.58–0.68	0.725–1.00	1,100
NOAA12 AVHRR2	1991/5/13	1	0.58–0.68	0.725–1.00	1,100
NOAA14 AVHRR2	1994/12/30	1	0.58–0.68	0.725–1.00	1,100
NOAA15 AVHRR3	1998/5/13	1	0.58–0.68	0.725–1.00	1,090
NOAA16 AVHRR3	2000/9/21	1	0.58–0.68	0.725–1.00	1,090
NOAA17 AVHRR3	2002/6/24	1	0.58–0.68	0.725–1.00	1,090
LANDSAT4 TM	1982/7/16	16	0.63–0.69	0.76–0.90	30
LANDSAT5 TM	1984/3/1	16	0.63–0.69	0.76–0.90	30
LANDSAT7 ETM+	1999/4/15	16	0.63–0.69	0.76–0.90	30
LANDSAT4 MSS	1982/7/16	16	0.60–0.70	0.70–0.80	82
LANDSAT5 MSS	1984/3/1	16	0.60–0.70	0.70–0.80	82
SPOT1 HRV	1986/2/22	26	0.61–0.68	0.78–0.89	20
SPOT4 HRVIR	1998/3/24	26	0.61–0.68	0.78–0.89	20
SPOT5 HRG	2002/5/4	26	0.61–0.68	0.78–0.89	10
CBERS02 CCD	2003/10/21	26	0.63–0.69	0.77–0.89	19.50
CBERS02B CCD	2007/9/19	26	0.63–0.69	0.77–0.89	20
HJ-1A CCD1	2008/9/6	4	0.63–0.69	0.76–0.90	30
HJ-1A CCD2	2008/9/6	4	0.63–0.69	0.76–0.90	30
HJ-1B CCD1	2008/9/6	4	0.63–0.69	0.76–0.90	30
HJ-1B CCD2	2008/9/6	4	0.63–0.69	0.76–0.90	30
IKONOS	1999/9/24	3–4	0.632–0.698	0.757–0.853	4
QuickBird	2001/10/18	1–3.5	0.63–0.69	0.76–0.90	2.44
Terra ASTER	1999/12/18	1–2	0.63–0.69	0.76–0.86	15
ALOS AVNIR2	2006/1/24	46	0.61–0.69	0.76–0.89	10
KOMPSAT2	2006/7/28	14	0.63–0.69	0.76–0.90	4
GEOEYE_1	2008/9/6	2–3	0.655–0.690	0.78–0.92	1.65
MODIS	1999/12/18	1–2	0.62–0.67	0.841–0.876	250–1,000

**Table 2.** Statistical description of the simulated red reflectance, the relative percentage difference (RPD) and the absolute percentage difference (APD) with respect to Terra MODIS. *N* = 447.

**Sensor**	**Reflectance**				**Relative Percentage Difference (%)**				**Absolute Percentage Difference (%)**				**t**	**P-value**
		
	**MIN**	**MAX**	**Mean**	**SD**	**MIN**	**MAX**	**Mean**	**SD**	**MIN**	**MAX**	**Mean**	**SD**		
NOAA7 AVHRR2	0.0169	0.1405	0.0415	0.0175	1.12	41.95	20.66	8.65	1.12	41.95	20.66	8.65	−79.28	0.000
NOAA8 AVHRR1	0.0158	0.1403	0.0409	0.0177	1.20	34.96	18.11	7.00	1.20	34.96	18.11	7.00	−76.79	0.000
NOAA9 AVHRR2	0.0180	0.1433	0.0438	0.0178	2.18	54.56	27.96	10.79	2.18	54.56	27.96	10.79	−87.05	0.000
NOAA10 AVHRR1	0.0157	0.1399	0.0405	0.0176	0.97	33.61	17.10	6.92	0.97	33.61	17.10	6.92	−71.78	0.000
NOAA11 AVHRR2	0.0185	0.1432	0.0441	0.0176	2.18	57.70	29.04	11.57	2.18	57.70	29.04	11.57	−89.34	0.000
NOAA12 AVHRR2	0.0204	0.1438	0.0454	0.0174	2.49	68.32	33.84	14.07	2.49	68.32	33.84	14.07	−93.21	0.000
NOAA14 AVHRR2	0.0193	0.1445	0.0453	0.0177	2.85	64.55	32.98	12.75	2.85	64.55	32.98	12.75	−93.03	0.000
NOAA15 AVHRR3	0.0150	0.1370	0.0386	0.0173	0.40	22.35	10.93	4.79	0.40	22.35	10.93	4.79	−71.57	0.000
NOAA16 AVHRR3	0.0143	0.1367	0.0381	0.0174	−0.02	17.82	9.07	3.84	0.02	17.82	9.07	3.84	−64.27	0.000
NOAA17 AVHRR3	0.0136	0.1357	0.0372	0.0175	−0.43	11.68	5.99	2.53	0.11	11.68	5.99	2.53	−56.16	0.000
LANDSAT4 TM	0.0131	0.1335	0.0357	0.0173	−2.81	3.80	1.11	1.19	0.01	3.80	1.36	0.90	−13.13	0.000
LANDSAT5 TM	0.0131	0.1334	0.0357	0.0173	−3.04	3.65	0.95	1.22	0.00	3.65	1.28	0.87	−10.59	0.000
LANDSAT7 ETM+	0.0120	0.1315	0.0336	0.0171	−9.73	0.19	−5.60	2.03	0.09	9.73	5.61	2.03	49.60	0.000
LANDSAT4 MSS	0.0144	0.1371	0.0387	0.0177	1.21	19.29	10.72	3.45	1.21	19.29	10.72	3.45	−85.51	0.000
LANDSAT5 MSS	0.0139	0.1361	0.0378	0.0176	0.87	13.67	7.66	2.37	0.87	13.67	7.66	2.37	−80.65	0.000
SPOT1 HRV	0.0135	0.1361	0.0373	0.0177	1.20	10.28	6.10	1.90	1.20	10.28	6.10	1.90	−61.00	0.000
SPOT4 HRVIR	0.0157	0.1373	0.0396	0.0174	1.06	28.13	14.13	5.58	1.06	28.13	14.13	5.58	−90.74	0.000
SPOT5 HRG	0.0134	0.1344	0.0365	0.0174	−0.08	7.57	3.75	1.47	0.03	7.57	3.75	1.47	−61.51	0.000
CBERS02 CCD	0.0150	0.1362	0.0387	0.0174	0.57	21.89	10.97	4.37	0.57	21.89	10.97	4.37	−77.88	0.000
CBERS02B CCD	0.0180	0.1400	0.0427	0.0176	2.39	46.21	24.07	9.15	2.39	46.21	24.07	9.15	−88.26	0.000
HJ–1A CCD1	0.0147	0.1344	0.0372	0.0171	−2.85	15.65	6.27	3.68	0.01	15.65	6.32	3.59	−43.63	0.000
HJ–1A CCD2	0.0153	0.1373	0.0395	0.0177	1.39	25.42	13.25	4.71	1.39	25.42	13.25	4.71	−79.21	0.000
HJ–1B CCD1	0.0135	0.1339	0.0362	0.0173	−1.63	6.62	2.81	1.55	0.01	6.62	2.85	1.48	−37.94	0.000
HJ–1B CCD2	0.0150	0.1363	0.0388	0.0175	0.98	21.63	11.27	4.24	0.98	21.63	11.27	4.24	−82.23	0.000
IKONOS	0.0221	0.1421	0.0460	0.0169	2.13	77.73	36.30	16.66	2.13	77.73	36.30	16.66	−87.07	0.000
QuickBird	0.0165	0.1362	0.0393	0.0170	0.19	30.98	13.76	6.85	0.19	30.98	13.76	6.85	−11.24	0.000
Terra ASTER	0.0134	0.1346	0.0366	0.0175	−0.68	7.68	3.91	1.53	0.22	7.68	3.92	1.51	−46.13	0.000
ALOS AVNIR2	0.0134	0.1346	0.0367	0.0175	0.32	8.14	4.25	1.43	0.32	8.14	4.25	1.43	−75.25	0.000
KOMPSAT2	0.0123	0.1322	0.0343	0.0172	−6.54	1.20	−3.58	1.57	0.02	6.54	3.62	1.47	36.62	0.000
GEOEYE_1	0.0111	0.1283	0.0313	0.0166	−21.02	−0.88	−12.67	4.12	0.88	21.02	12.67	4.12	50.73	0.000
MODIS	0.0128	0.1337	0.0354	0.0174	∼	∼	∼	∼	∼	∼	∼	∼	∼	∼

**Table 3.** Parameters of the exponential and quadratic fit to the relative spectral correction for simulated reflectance in the red channel. Quadratic equation: *y* = c + b1*x* + b2*x*^2^; Exponential equation: *y* = c·exp(b1*x*), y denotes RPD in the red reflectance, x denotes NDVI for particular sensor.

**Sensor**	**Equation**	**Model Summary**					**Parameter Estimates**		
	
		**R Square**	**F**	**df1**	**df2**	**Sig.**	**c**	**b1**	**b2**
NOAA7 AVHRR2	Exponential	0.765	1,445.053	1	445	0.000	0.016	3.384	
NOAA8 AVHRR1	Exponential	0.727	1,186.784	1	445	0.000	0.018	3.015	
NOAA9 AVHRR2	Exponential	0.810	1,898.691	1	445	0.000	0.027	3.134	
NOAA10 AVHRR1	Exponential	0.678	935.802	1	445	0.000	0.015	3.137	
NOAA11 AVHRR2	Exponential	0.838	2,294.369	1	445	0.000	0.025	3.308	
NOAA12 AVHRR2	Exponential	0.892	3,657.223	1	445	0.000	0.024	3.615	
NOAA14 AVHRR2	Exponential	0.858	2,680.440	1	445	0.000	0.031	3.201	
NOAA15 AVHRR3	Exponential	0.730	1,201.323	1	445	0.000	0.006	3.695	
NOAA16 AVHRR3	Quadratic	0.723	578.057	2	444	0.000	0.100	−0.384	0.478
NOAA17 AVHRR3	Quadratic	0.619	360.566	2	444	0.000	0.057	−0.207	0.270
LANDSAT4 TM	Quadratic	0.259	77.709	2	444	0.000	0.010	−0.062	0.080
LANDSAT5 TM	Quadratic	0.201	56.011	2	444	0.000	0.007	–0.051	0.069
LANDSAT7 ETM+	Quadratic	0.520	240.495	2	444	0.000	-0.051	0.146	−0.188
LANDSAT4 MSS	Exponential	0.865	2,841.229	1	445	0.000	0.012	2.801	
LANDSAT5 MSS	Exponential	0.830	2,177.267	1	445	0.000	0.010	2.645	
SPOT1 HRV	Exponential	0.576	605.598	1	445	0.000	0.010	2.280	
SPOT4 HRVIR	Exponential	0.940	6,976.599	1	445	0.000	0.008	3.729	
SPOT5 HRG	Quadratic	0.741	635.947	2	444	0.000	0.025	−0.098	0.145
CBERS_02 CCD	Exponential	0.850	2,521.980	1	445	0.000	0.006	3.793	
CBERS-02B CCD	Exponential	0.901	4,038.328	1	445	0.000	0.017	3.475	
HJ-1A CCD1	Quadratic	0.775	765.162	2	444	0.000	0.115	−0.513	0.565
HJ-1A CCD2	Exponential	0.783	1,603.250	1	445	0.000	0.012	3.069	
HJ-1B CCD1	Quadratic	0.646	404.794	2	444	0.000	0.030	−0.142	0.176
HJ-1B CCD2	Exponential	0.880	3,254.515	1	445	0.000	0.008	3.457	
IKONOS	Exponential	0.962	11,172.667	1	445	0.000	0.013	4.514	
QuickBird	Exponential	0.963	11,614.356	1	445	0.000	0.003	5.059	
Terra ASTER	Quadratic	0.474	199.887	2	444	0.000	0.011	−0.027	0.081
ALOS AVNIR2	Exponential	0.817	1,983.879	1	445	0.000	0.004	2.977	
KOMPSAT2	Quadratic	0.348	118.723	2	444	0.000	−0.036	0.101	−0.126
GEOEYE_1	Quadratic	0.469	196.459	2	444	0.000	−0.103	0.242	−0.332

**Table 4.** Statistical description of the simulated NIR reflectance, the relative percentage difference (RPD) and the absolute percentage difference (APD) with respect to Terra MODIS. *N* = 447.

**Sensor**	**Reflectance**				**Relative Percentage Difference (%)**				**Absolute Percentage Difference (%)**				**t**	**P-value**
		
	**MIN**	**MAX**	**Mean**	**SD**	**MIN**	**MAX**	**Mean**	**SD**	**MIN**	**MAX**	**Mean**	**SD**		
NOAA7 AVHRR2	0.0838	0.4614	0.2793	0.0674	−11.79	43.94	−7.42	2.73	3.72	43.94	7.61	2.12	66.22	0.000
NOAA8 AVHRR1	0.0851	0.4542	0.2757	0.0659	−11.88	41.75	−8.52	2.79	4.02	41.75	8.71	2.13	62.69	0.000
NOAA9 AVHRR2	0.0852	0.4601	0.2787	0.0669	−10.53	43.54	−7.56	2.74	3.54	43.54	7.76	2.12	63.76	0.000
NOAA10 AVHRR1	0.0858	0.4687	0.2830	0.0684	−9.71	46.27	−6.17	2.70	2.81	46.27	6.38	2.17	65.50	0.000
NOAA11 AVHRR2	0.0853	0.4596	0.2784	0.0668	−10.69	43.37	−7.66	2.74	3.56	43.37	7.86	2.12	63.48	0.000
NOAA12 AVHRR2	0.0854	0.4624	0.2798	0.0673	−10.29	44.29	−7.20	2.73	3.29	44.29	7.39	2.13	63.86	0.000
NOAA14 AVHRR2	0.0833	0.4647	0.2809	0.0681	−12.29	44.95	−6.93	2.75	3.51	44.95	7.14	2.17	67.86	0.000
NOAA15 AVHRR3	0.0864	0.4754	0.2864	0.0695	−8.71	48.30	−5.08	2.68	2.22	48.30	5.30	2.22	67.33	0.000
NOAA16 AVHRR3	0.0854	0.4755	0.2863	0.0697	−9.70	48.28	−5.15	2.70	2.41	48.28	5.36	2.24	69.75	0.000
NOAA17 AVHRR3	0.0857	0.4765	0.2868	0.0699	−9.38	48.62	−4.98	2.69	2.33	48.62	5.19	2.25	70.20	0.000
LANDSAT4 TM	0.0964	0.5003	0.3003	0.0728	−2.44	56.66	−0.40	2.85	0.00	56.66	0.98	2.71	16.60	0.000
LANDSAT5 TM	0.0967	0.5003	0.3002	0.0727	−2.57	56.64	−0.42	2.87	0.00	56.64	1.02	2.71	16.45	0.000
LANDSAT7 ETM+	0.0972	0.5000	0.3000	0.0726	−2.93	56.62	−0.47	2.91	0.00	56.62	1.12	2.73	15.91	0.000
LANDSAT4 MSS	0.0972	0.4998	0.2997	0.0726	−3.17	56.53	−0.58	2.93	0.00	56.53	1.20	2.74	17.30	0.000
LANDSAT5 MSS	0.0969	0.5001	0.2999	0.0727	−2.91	56.58	−0.53	2.90	0.00	56.58	1.12	2.73	17.33	0.000
SPOT1 HRV	0.0995	0.4977	0.2983	0.0718	−4.83	55.98	−0.99	3.20	0.01	55.98	1.79	2.83	18.10	0.000
SPOT4 HRVIR	0.0968	0.4996	0.2998	0.0726	−2.95	56.45	−0.57	2.90	0.00	56.45	1.15	2.72	18.31	0.000
SPOT5 HRG	0.0972	0.5013	0.3008	0.0728	−2.33	56.94	−0.23	2.88	0.01	56.94	0.94	2.73	11.99	0.000
CBERS02 CCD	0.0963	0.4915	0.2955	0.0711	−4.46	53.95	−1.92	2.92	0.00	53.95	2.28	2.65	36.72	0.000
CBERS02B CCD	0.0960	0.5005	0.3006	0.0729	−1.95	56.67	−0.33	2.80	0.00	56.67	0.84	2.69	17.87	0.000
HJ-1A CCD1	0.0973	0.4962	0.2978	0.0719	−4.09	55.47	−1.20	2.98	0.01	55.47	1.70	2.73	25.66	0.000
HJ-1A CCD2	0.0976	0.4967	0.2980	0.0719	−4.23	55.64	−1.11	3.02	0.03	55.64	1.68	2.75	23.09	0.000
HJ-1B CCD1	0.0975	0.4971	0.2982	0.0720	−4.14	55.74	−1.06	3.01	0.00	55.74	1.62	2.75	22.44	0.000
HJ-1B CCD2	0.0969	0.4965	0.2979	0.0720	−4.07	55.52	−1.17	2.97	0.02	55.52	1.65	2.73	25.11	0.000
IKONOS	0.0961	0.4610	0.2782	0.0656	−12.12	44.60	−7.46	3.52	0.08	44.60	7.69	2.98	50.97	0.000
QuickBird	0.0965	0.4733	0.2852	0.0678	−9.31	48.41	−5.23	3.29	0.07	48.41	5.50	2.83	13.74	0.000
Terra ASTER	0.0999	0.4923	0.2952	0.0708	−6.81	54.41	−1.97	3.43	0.00	54.41	2.64	2.93	24.30	0.000
ALOS AVNIR2	0.0983	0.4959	0.2974	0.0717	−4.98	55.42	−1.29	3.14	0.01	55.42	1.92	2.80	22.39	0.000
KOMPSAT2	0.0950	0.4932	0.2964	0.0717	−4.40	54.49	−1.67	2.91	0.01	54.49	1.99	2.70	32.76	0.000
GEOEYE_1	0.0963	0.5009	0.3005	0.0729	−2.25	56.78	−0.34	2.84	0.00	56.78	0.91	2.71	15.76	0.000
MODIS	0.0927	0.5023	0.3019	0.0737	∼	∼	∼	∼	∼	∼	∼	∼	∼	∼

**Table 5.** Parameters of polynomial fit to the relative spectral correction for simulated reflectance in NIR channel. Equation: *y* = c + b1*x* + b2*x*^2^, y denotes RPD in the NIR reflectance, x denotes NDVI for particular sensor.

**Sensor**	**Equation**	**Model Summary**					**Parameter Estimates**		
	
		**R Square**	**F**	**df1**	**df2**	**Sig.**	**c**	**b1**	**b2**
NOAA7 AVHRR2	Quadratic	0.524	244.409	2	444	0.000	−0.127	0.256	−0.246
NOAA8 AVHRR1	Quadratic	0.688	489.556	2	444	0.000	−0.108	0.197	−0.223
NOAA9 AVHRR2	Quadratic	0.637	390.357	2	444	0.000	−0.105	0.203	−0.219
NOAA10 AVHRR1	Quadratic	0.499	221.154	2	444	0.000	−0.104	0.205	−0.197
NOAA11 AVHRR2	Quadratic	0.640	393.833	2	444	0.000	−0.106	0.203	−0.222
NOAA12 AVHRR2	Quadratic	0.590	319.582	2	444	0.000	−0.106	0.209	−0.223
NOAA14 AVHRR2	Quadratic	0.455	185.492	2	444	0.000	−0.134	0.284	−0.264
NOAA15 AVHRR3	Quadratic	0.413	156.106	2	444	0.000	−0.096	0.190	−0.170
NOAA16 AVHRR3	Quadratic	0.371	131.143	2	444	0.000	−0.110	0.223	−0.189
NOAA17 AVHRR3	Quadratic	0.359	124	2	444	0.000	−0.106	0.213	−0.178
LANDSAT4 TM	Quadratic	0.214	60.374	2	444	0.000	0.053	−0.151	0.096
LANDSAT5 TM	Quadratic	0.213	59.909	2	444	0.000	0.056	−0.161	0.102
LANDSAT7 ETM+	Quadratic	0.198	54.916	2	444	0.000	0.064	−0.181	0.114
LANDSAT4 MSS	Quadratic	0.204	57.047	2	444	0.000	0.063	−0.188	0.122
LANDSAT5 MSS	Quadratic	0.205	57.289	2	444	0.000	0.058	−0.172	0.111
SPOT1 HRV	Quadratic	0.208	58.236	2	444	0.000	0.094	−0.275	0.174
SPOT4 HRVIR	Quadratic	0.208	58.321	2	444	0.000	0.057	−0.172	0.111
SPOT5 HRG	Quadratic	0.222	63.487	2	444	0.000	0.059	−0.164	0.104
CBERS_02 CCD	Quadratic	0.319	104.165	2	444	0.000	0.057	−0.185	0.106
CBERS-02B CCD	Quadratic	0.244	71.667	2	444	0.000	0.043	−0.129	0.084
HJ-1A CCD1	Quadratic	0.223	63.772	2	444	0.000	0.071	−0.219	0.138
HJ-1A CCD2	Quadratic	0.213	59.943	2	444	0.000	0.071	−0.218	0.139
HJ-1B CCD1	Quadratic	0.202	56.161	2	444	0.000	0.071	−0.213	0.134
HJ-1B CCD2	Quadratic	0.207	58.105	2	444	0.000	0.062	−0.196	0.123
IKONOS	Quadratic	0.411	154.853	2	444	0.000	0.084	−0.386	0.220
QuickBird	Quadratic	0.376	133.838	2	444	0.000	0.079	−0.306	0.167
Terra ASTER	Quadratic	0.199	55.035	2	444	0.000	0.108	−0.332	0.208
ALOS AVNIR2	Quadratic	0.200	55.513	2	444	0.000	0.083	−0.250	0.157
KOMPSAT2	Quadratic	0.193	52.950	2	444	0.000	0.042	−0.142	0.080
GEOEYE_1	Quadratic	0.198	54.767	2	444	0.000	0.054	−0.150	0.094

**Table 6.** Statistical description of the simulated NDVI, the relative percentage difference (RPD) and the absolute percentage difference (APD) with respect to Terra MODIS. *N* = 447.

**Sensor**	**NDVI**				**Relative Percentage Difference (%)**				**Absolute Percentage Difference (%)**				**t**	**P-value**
		
	**MIN**	**MAX**	**Mean**	**SD**	**MIN**	**MAX**	**Mean**	**SD**	**MIN**	**MAX**	**Mean**	**SD**		
NOAA7 AVHRR2	0.1268	0.8900	0.7271	0.1372	−28.39	2.11	−6.72	3.13	2.11	28.39	6.73	3.11	127.31	0.000
NOAA8 AVHRR1	0.1289	0.8943	0.7284	0.1380	−27.19	2.51	−6.56	3.05	2.51	27.19	6.57	3.02	124.86	0.000
NOAA9 AVHRR2	0.1259	0.8826	0.7147	0.1370	−28.87	0.85	−8.37	3.31	0.85	28.87	8.37	3.30	149.47	0.000
NOAA10 AVHRR1	0.1334	0.8984	0.7354	0.1375	−24.67	3.19	−5.61	2.80	2.74	24.67	5.62	2.77	109.36	0.000
NOAA11 AVHRR2	0.1252	0.8788	0.7131	0.1360	−29.31	0.52	−8.56	3.18	0.52	29.31	8.56	3.17	170.66	0.000
NOAA12 AVHRR2	0.1240	0.8686	0.7071	0.1341	−29.94	−0.39	−9.29	2.99	0.39	29.94	9.29	2.99	225.79	0.000
NOAA14 AVHRR2	0.1147	0.8762	0.7081	0.1374	−35.24	0.08	−9.29	3.79	0.08	35.24	9.29	3.79	157.31	0.000
NOAA15 AVHRR3	0.1402	0.9047	0.7481	0.1362	−20.81	4.48	−3.85	2.09	2.06	20.81	3.87	2.05	114.52	0.000
NOAA16 AVHRR3	0.1365	0.9086	0.7506	0.1376	−22.90	4.91	−3.57	2.37	1.63	22.90	3.59	2.34	83.79	0.000
NOAA17 AVHRR3	0.1385	0.9132	0.7559	0.1379	−21.76	5.56	−2.87	2.21	1.13	21.76	2.89	2.18	70.05	0.000
LANDSAT4 TM	0.1821	0.9210	0.7735	0.1317	−2.79	7.07	−0.21	0.87	0.00	7.07	0.59	0.67	10.97	0.000
LANDSAT5 TM	0.1824	0.9211	0.7737	0.1316	−2.92	7.10	−0.18	0.92	0.00	7.10	0.60	0.71	9.42	0.000
LANDSAT7 ETM+	0.1874	0.9274	0.7845	0.1312	−1.59	8.16	1.28	1.28	0.01	8.16	1.34	1.22	−32.04	0.000
LANDSAT4 MSS	0.1746	0.9129	0.7571	0.1329	−5.46	5.62	−2.44	0.84	0.36	5.62	2.47	0.76	85.76	0.000
LANDSAT5 MSS	0.1759	0.9163	0.7621	0.1331	−4.39	6.15	−1.79	0.73	0.55	6.15	1.82	0.65	74.04	0.000
SPOT1 HRV	0.1766	0.9175	0.7637	0.1328	−4.18	6.40	−1.55	0.85	0.02	6.40	1.62	0.69	56.02	0.000
SPOT4 HRVIR	0.1749	0.9059	0.7526	0.1310	−5.75	4.78	−3.00	0.79	0.36	5.75	3.02	0.70	96.30	0.000
SPOT5 HRG	0.1811	0.9191	0.7694	0.1319	−2.94	6.71	−0.77	0.76	0.01	6.71	0.92	0.56	34.46	0.000
CBERS02 CCD	0.1753	0.9081	0.7548	0.1317	−5.99	5.10	−2.71	0.92	0.34	5.99	2.73	0.85	77.44	0.000
CBERS02B CCD	0.1687	0.8932	0.7375	0.1312	−9.65	3.12	−5.03	1.22	3.12	9.65	5.05	1.16	114.15	0.000
HJ-1A CCD1	0.1815	0.9108	0.7644	0.1300	−4.88	5.86	−1.37	1.11	0.01	5.86	1.59	0.77	36.50	0.000
HJ-1A CCD2	0.1730	0.9069	0.7523	0.1324	−7.29	4.90	−3.07	1.10	0.47	7.29	3.09	1.03	72.48	0.000
HJ-1B CCD1	0.1806	0.9179	0.7696	0.1314	−3.81	6.69	−0.72	0.96	0.00	6.69	0.97	0.70	24.87	0.000
HJ-1B CCD2	0.1737	0.9088	0.7556	0.1319	−6.24	5.25	−2.62	0.93	0.53	6.24	2.64	0.86	74.00	0.000
IKONOS	0.1579	0.8571	0.7043	0.1265	−15.21	−1.19	−9.32	1.47	1.19	15.21	9.32	1.47	132.43	0.000
QuickBird	0.1715	0.8947	0.7447	0.1296	−7.62	3.69	−4.00	0.94	0.85	7.62	4.01	0.87	13.00	0.000
Terra ASTER	0.1787	0.9166	0.7658	0.1321	−6.15	6.49	−1.24	1.27	0.01	6.49	1.43	1.04	27.85	0.000
ALOS AVNIR2	0.1784	0.9178	0.7665	0.1323	−4.14	6.55	−1.16	0.88	0.05	6.55	1.27	0.71	39.41	0.000
KOMPSAT2	0.1794	0.9240	0.7788	0.1323	−2.89	7.67	0.48	1.00	0.00	7.67	0.75	0.82	−11.99	0.000
GEOEYE_1	0.1981	0.9326	0.7973	0.1288	−0.56	14.16	3.10	2.31	0.01	14.16	3.11	2.31	−45.04	0.000
MODIS	0.1770	0.9237	0.7756	0.1332	∼	∼	∼	∼	∼	∼	∼	∼	∼	∼

**Table 7.** Parameters of the polynomial fit to the relative spectral correction for the NDVI. Equation: *y* = c + b1*x* + b2*x*^2^, y denotes RPD in the NDVI, x denotes NDVI for particular sensor.

**Sensor**	**Equation**	**Model Summary**					**Parameter Estimates**		
	
		**R Square**	**F**	**df1**	**df2**	**Sig.**	**c**	**b1**	**b2**
NOAA7 AVHRR2	Quadratic	0.879	1,616.536	2	444	0.000	−0.324	0.604	−0.333
NOAA8 AVHRR1	Quadratic	0.898	1,961.791	2	444	0.000	−0.299	0.515	−0.258
NOAA9 AVHRR2	Quadratic	0.888	1,767.126	2	444	0.000	−0.324	0.525	−0.255
NOAA10 AVHRR1	Quadratic	0.870	1,479.434	2	444	0.000	−0.282	0.516	−0.274
NOAA11 AVHRR2	Quadratic	0.890	1,793.728	2	444	0.000	−0.322	0.524	−0.261
NOAA12 AVHRR2	Quadratic	0.892	1,840.831	2	444	0.000	−0.321	0.518	−0.267
NOAA14 AVHRR2	Quadratic	0.895	1,888.605	2	444	0.000	−0.381	0.666	−0.353
NOAA15 AVHRR3	Quadratic	0.863	1,400.234	2	444	0.000	−0.227	0.448	−0.254
NOAA16 AVHRR3	Quadratic	0.869	1,473.320	2	444	0.000	−0.251	0.516	−0.295
NOAA17 AVHRR3	Quadratic	0.874	1,537	2	444	0.000	−0.234	0.492	−0.282
LANDSAT4 TM	Quadratic	0.447	179.534	2	444	0.000	0.079	−0.220	0.146
LANDSAT5 TM	Quadratic	0.444	176.978	2	444	0.000	0.084	−0.233	0.154
LANDSAT7 ETM+	Quadratic	0.535	255.479	2	444	0.000	0.120	−0.250	0.140
LANDSAT4 MSS	Quadratic	0.640	394.503	2	444	0.000	0.026	−0.210	0.184
LANDSAT5 MSS	Quadratic	0.603	336.802	2	444	0.000	0.031	−0.191	0.161
SPOT1 HRV	Quadratic	0.518	238.856	2	444	0.000	0.060	−0.260	0.206
SPOT4 HRVIR	Quadratic	0.548	269.560	2	444	0.000	0.037	−0.241	0.196
SPOT5 HRG	Quadratic	0.523	243.138	2	444	0.000	0.072	−0.237	0.167
CBERS_02 CCD	Quadratic	0.437	172.178	2	444	0.000	0.040	−0.248	0.204
CBERS-02B CCD	Quadratic	0.551	272.206	2	444	0.000	−0.014	−0.206	0.205
HJ-1A CCD1	Quadratic	0.410	154.517	2	444	0.000	0.093	−0.300	0.204
HJ-1A CCD2	Quadratic	0.488	211.672	2	444	0.000	0.038	−0.276	0.237
HJ-1B CCD1	Quadratic	0.422	162.398	2	444	0.000	0.086	−0.268	0.186
HJ-1B CCD2	Quadratic	0.419	160.089	2	444	0.000	0.037	−0.237	0.197
IKONOS	Quadratic	0.580	307.056	2	444	0.000	−0.026	−0.332	0.325
QuickBird	Quadratic	0.448	180.278	2	444	0.000	0.041	−0.286	0.231
Terra ASTER	Quadratic	0.346	117.496	2	444	0.000	0.105	−0.368	0.272
ALOS AVNIR2	Quadratic	0.441	175.009	2	444	0.000	0.074	−0.272	0.203
KOMPSAT2	Quadratic	0.244	71.633	2	444	0.000	0.068	−0.160	0.099
GEOEYE_1	Quadratic	0.568	291.870	2	444	0.000	0.193	−0.313	0.133
